# Supplementary material for: Neutralizing Antibodies against the SARS-CoV-2 Ancestral Strain and Omicron BA.1 Subvariant in Dogs and Cats in Mexico
Source: Pathogens. 2023 Jun 16;12(6):835. doi: 10.3390/pathogens12060835 (PMC10303733; doi:10.3390/pathogens12060835)
Supplement: Supplementary file 1 [file pathogens-12-00835-s001.zip › pathogens-2430676-supplementary.pdf]

Supplementary Table S1. 2019-Novel Coronavirus (2019-nCoV) Real-Time RT–qPCR panel primer and probes.

Name of the Primer	Description of the Primer	Oligonucleotide sequence (5’>3’)
2019- nCoV_N1-F	2019-nCoV_N1 Forward Primer	GAC CCC AAA ATC AGC GAA AT
2019-nCoV_N1-R	2019-nCoV_N1 Reverse Primer	TCT GGT TAC TGC CAG TTG AAT CTG
2019-nCoV_N1-P	2019-nCoV_N1 Probe	FAM-ACC CCG CAT TAC GTT TGG TGG ACC-BHQ1
2019-nCoV_N1-P	2019-nCoV_N1 Probe	FAM-ACC CCG CAT /ZEN/ TAC GTT TGG TGG ACC-3IABkFQ
2019-nCoV_N2-F	2019-nCoV_N2 Forward Primer	TTA CAA ACA TTG GCC GCA AA
2019-nCoV_N2-R	2019-nCoV_N2 Reverse Primer	GCG CGA CAT TCC GAA GAA
2019-nCoV_N2-P	2019-nCoV_N2 Probe	FAM-ACA ATT TGC CCC CAG CGC TTC AG-BHQ1
2019-nCoV_N2-P	2019-nCoV_N2 Probe	FAM-ACA ATT TGC /ZEN/ CCC CAG CGC TTC AG-3IABkFQ
RP-F	RNase P Forward Primer	AGA TTT GGA CCT GCG AGC G
RP-R	RNase P Reverse Primer	GAG CGG CTG TCT CCA CAA GT
RP-P	RNase P Probe	FAM – TTC TGA CCT GAA GGC TCT GCG CG – BHQ-1
RP-P	RNase P Probe	FAM-TTC TGA CCT /ZEN/ GAA GGC TCT GCG CG-3IABkFQ

Supplementary Table S2. Neutralizing antibodies against Ancestral strain and Omicron BA.1 subvariant in the positive control samples

Positive controls	Antisera origin	PRNT90 Ancestral strain	MN Ancestral strain	PRNT90 Ómicron (BA.1)	MN Ómicron (BA.1)
CPALBS32021014	Ancestral strain	1:40	1:20	1:20	1:10
CPALBS32022011	Ómicron (BA.1)	1:80	1:40	1:80	1:40
CPALBS32021014	Ancestral strain	1:20	1:20	1:20	1:10
CPALBS32022011	Ómicron (BA.1)	1:40	1:20	1:40	1:20
CPALBS32021014	Ancestral strain	1:40	1:20	1:20	1:10
CPALBS32022011	Ómicron (BA.1)	1:80	1:40	1:80	1:40
CPALBS32021014	Ancestral strain	1:40	1:20	1:20	1:10
CPALBS32022011	Ómicron (BA.1)	1:80	1:40	1:80	1:40
CPALBS32021014	Ancestral strain	1:20	1:10	1:20	1:10
CPALBS32022011	Ómicron (BA.1)	1:80	1:40	1:80	1:40
